# Maintaining sexual identity in adult flies is an ongoing commitment

**DOI:** 10.1371/journal.pbio.3003530

**Published:** 2025-12-19

**Authors:** Artyom Kopp

**Affiliations:** Department of Evolution and Ecology, University of California – Davis, Davis, California, United States of America

## Abstract

In the gonad, sex is not an irrevocable commitment and requires active maintenance. A new study in PLOS Biology elucidates the molecular mechanisms that preserve sexual identity in the adult *Drosophila* testis.

We normally think of animal sex, as defined by the presence of ovaries in females and testes in males, as an irreversible developmental commitment, determined by genetic or environmental factors during embryogenesis and fixed for the duration of adult life. However, it is becoming increasingly clear that continuous sex maintenance is necessary to preserve this commitment: even after testes or ovaries differentiate, their fate must be actively preserved rather than being irrevocably fixed. The most dramatic examples are found in fish that switch from a fully functional breeding male to an equally fertile female, or vice versa, in response to social cues [1]. In fish species where sex is normally permanent, such as zebrafish and medaka, a variety of disturbances, from oocyte depletion to reduced estrogen signaling to starvation, can induce female-to-male transformation during adult life [2,3]. Even in mammals, sex is not fully locked in at the end of embryonic development. Mammalian gonad is bipotential, with the male versus female decision being shaped by active competition between a testis-promoting gene network involving the transcription factors *Sry*, *Sox9*, and *Dmrt1*, and an ovary-promoting network centered on *Foxl2* [4,5]. In mice, the loss of *Dmrt1* in the adult testis reprograms the gonad toward an ovarian identity [5]; conversely, loss of *Foxl2* in the adult ovary triggers the reciprocal switch toward testis [4]. These experiments show that the male and female gene networks must continually repress the alternate state, and that maintaining gonadal sex is an active process distinct from the initial sex determination. Work in *Drosophila* suggests that, for all the differences between vertebrates and insects, insect sex is also a matter of ongoing maintenance rather than irreversible commitment [6,7]. A new paper by Harsh and colleagues published in this issue of PLOS Biology offers a deeper insight into how this maintenance plays out [8].

In *Drosophila*, the germline has an intrinsic sexual identity independent of the somatic sex, and proper gonad development requires close communication between germline and somatic cells [9]. In the testis, somatic cyst stem cells (CySCs) and their progeny are central to this communication. A key role is played by the *chinmo* transcription factor acting downstream from JAK-STAT signaling [6]. *chinmo* is not normally expressed in the ovary; in the adult testis, however, loss of *chinmo* in CySCs causes these cells to adopt a female-like developmental program, leading to mismatched germline-soma communication and sterility. Female somatic development in *Drosophila* and most other insects is promoted by the female-specific isoform of the splicing regulator *transformer* (*traF*), while male somatic development is controlled by the male-specific isoform of the transcription factor *doublesex* (*dsxM*), which is produced in the absence of *traF* [10]. *chinmo* expression in the testis under the control of the JAK-STAT pathway prevents the production of *traF* in CySCs, supporting continued expression of *dsxM* and maintaining the male sexual identity of these cells. When *chinmo* levels fall, CySCs begin to express *traF* and to lose *dsxM*, switching them from the male to the female somatic program [6,7]. Thus, while the molecular pathways involved in sex determination are quite different between vertebrates and insects, the overall logic is similar: an active competition between male- and female-promoting developmental programs necessitates ongoing maintenance of sexual commitment.

The new study [8] elucidates a key mechanism by which *chinmo* acts to maintain male sexual identity in the gonad (Fig 1). It shows that *chinmo* promotes the expression of several microRNAs that post-transcriptionally repress *zelda* (*zld*), a pioneer transcription factor that functions to open compacted chromatin and to license regulatory elements for activation by other transcription factors [11]. When *chinmo* is lost, this miRNA barrier collapses, and increasing levels of Zld protein in CySCs activate the transcription of multiple genes that are normally repressed in the testis. Of these, the two key effectors are the RNA-binding protein *qkr58E-2* and *EcR*, the receptor for the hormone ecdysone that controls many aspects of insect development. *qkr58E-2* promotes female-specific splicing of *transformer* to produce *traF*, which then redirects the *dsx* pre-mRNA to the female isoform (*dsxF*), thereby switching the sex determination cascade from the male to the female state. In parallel, *EcR* reinforces the female transcriptional program by upregulating genes associated with ovarian development. Importantly, depleting *zld* in *chinmo*-deficient CySCs suppresses feminization, demonstrating that *zld* is the proximate driver of sexual transformation downstream of *chinmo*. Conversely, ectopic *zld* in an otherwise wild-type male soma induces *traF*, elevates *EcR*, and feminizes the tissue, highlighting *zld*’s pioneer activity in enabling female development. Together, the *zld* – *qkr58E-2* – *traF* – *dsxF* and the *zld* – *EcR* pathways form a feed-forward module that overrides the male state, resulting in the failure of sex maintenance and in male-to-female somatic sex reversal. In parallel, *EcR* represses *chinmo*, stabilizing the departure from the male-specific program (Fig 1).

**Fig 1:**
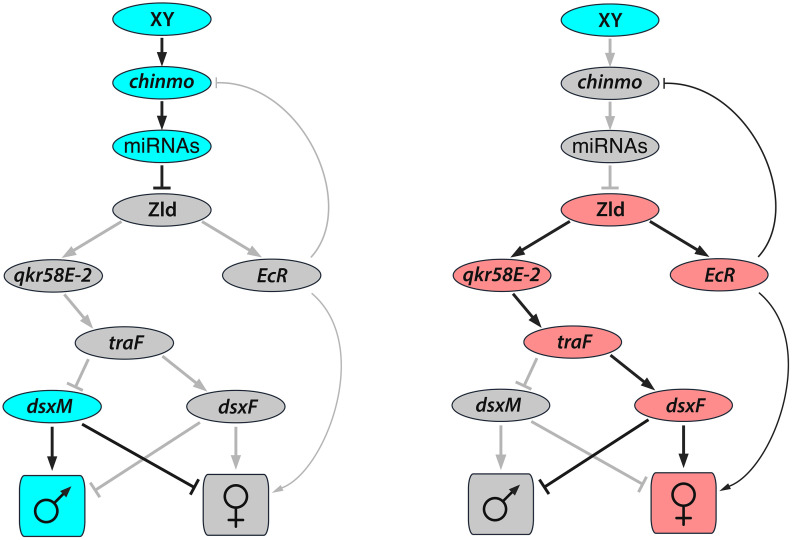
Somatic sex maintenance in the *Drosophila* testis. Left: in XY adults, *chinmo* prevents Zld protein expression, keeping *traF* off and allowing *dsxM* to maintain the male fate (blue) while the female pathway is switched off (gray). Right: loss of *chinmo* leads to the ectopic expression of *zld,* inducing *traF* and activating the female developmental program (red) in chromosomally male cells.

The study of Harsh and colleagues [8] points to pioneer factor-gated chromatin opening as a central point in the failure of sex maintenance. How general is this mechanism? Is it just a coincidence that one of the hundreds of *zelda* targets is one of the multiple RNA-binding proteins required for the splicing of *transformer*? Or is there a deeper principle in play? After all, pioneer transcription factors are the masters of developmental reprogramming [11]. Intriguingly, *Dmrt1* has been proposed to have a pioneer-like function in sex transformation in the mammalian testis [12], and sex transition in fish is accompanied by widespread chromatin changes [1]. Perhaps fruit flies and sex-changing reef fish have more in common than we think.

## Abbreviations

CySCs: somatic cyst stem cells
*dsxF*: dsxF female-specific isoform of the transcription factor doublesex
*dsxM*: male-specific isoform of the transcription factor doublesex
*traF*: female-specific isoform of the splicing regulator transformer
*zld*: zelda
